# The United Nations convention on rare diseases—A framework for research prioritization

**DOI:** 10.3389/fpubh.2025.1706653

**Published:** 2025-12-09

**Authors:** Mohammed Junaid, Jenny Downs, Tudor Groza, Timo Lassmann, Sue Baker, Kaila Stevens, Jake Keeffe, Dylan Gration, Amanda Newell, Jamie Duckers, Gareth Baynam

**Affiliations:** 1 University of Western Australia Dental School, Nedlands, WA, Australia; 2 The Kids Research Institute Australia, Nedlands, WA, Australia; 3 Curtin School of Allied Health, Curtin University, Perth, WA, Australia; 4 Perth Children's Hospital, Nedlands, WA, Australia; 5 Bioinformatics Institute, Agency for Science Technology and Research, Singapore, Singapore; 6 King Edward Memorial Hospital, Subiaco, WA, Australia; 7 NHS Wales Cardiff and Vale University Health Board, Cardiff, United Kingdom; 8 University of Western Australia, Perth, WA, Australia

**Keywords:** rare disease (RD), rare disease diagnosis, orphan drug, rare disease research, rare disease registry framework

## Introduction

Collectively, rare diseases (RD) are estimated to affect 3.5%−5.9% of the population, equating to approximately 263–446 million persons globally (1). Despite their diversity and prevalence, persons living with rare diseases (PLWRD) face many common challenges, such as lack of awareness across medical and general communities, diagnostic challenges, a poor evidence base for treatment, and difficulties achieving adequate healthcare (2).

On 16 December 2021, the United Nations (UN) General Assembly adopted the first-ever resolution on “Addressing the Challenges of Persons Living with a Rare Disease and their Families” (3, 4). The Resolution recognizes the specific needs and rights of this vulnerable population and calls for global action to improve their health and wellbeing, highlighting the importance of research and innovation as a key driver of scientific and social progress (4). In November 2023, the Resolution was updated, increasing emphasis on people with undiagnosed rare diseases (34). We examine the research needs for rare diseases considering the Resolution and make recommendations to address them. Existing literature and reports have predominantly focused on describing epidemiological patterns or reiterating the unmet needs of this population, without translating the 2021 United Nations (UN) Resolution into an actionable research or governance agenda (5, 6). This opinion piece conceptualizes application of the Resolution as a research governance instrument [Resolution-Informed Research Governance (RIRG)] linking human-rights obligations to empirical implementation pathways to bridge gaps between rhetoric and measurable impact. To facilitate this, we have grouped our recommendations thematically into four aspects: data, diagnosis, treatment, and participation (Table 1). We propose that the Resolution provides a unique research framework because it: (1) captures the global voice of PLWRD and their unmet needs, and (2) provides a clear and transparent way for Member States to fulfill their responsibilities, globally because all UN Member States endorsed the Resolution.

**Table 1:** A summary of research needs and recommendations.

**Aspect**	**Research needs**	**Recommendations**
Data	Lack of reliable and comparable data on rare diseases	1. Establish and support national and international registries and biobanks for rare diseases 2. Promote and facilitate data interoperability and accessibility 3. Enhance data literacy and capacity among stakeholders
Diagnosis	Delay and difficulty in obtaining a correct and timely diagnosis	1. Develop and implement national and international strategies and guidelines for rare disease diagnosis 2. Invest and innovate in diagnostic technologies and methods 3. Empower and educate patients and families
Treatment	Lack of effective and accessible treatments and therapies	1. Increase and diversify funding and incentives for rare disease research and development 2. Foster and facilitate translational and clinical research for rare diseases 3. Ensure and expand access and affordability of rare disease treatments and therapies
Participation	Marginalization and exclusion of persons living with a rare disease and their families from society	1. Raise and improve awareness and understanding of rare diseases among the public, the media, and the policymakers 2. Protect and promote the human rights and interests of persons living with a rare disease and their families 3. Support and strengthen the rare disease community and civil society

## Data

One of the main challenges for RD is the lack of reliable and comparable data on their epidemiology, natural history, and impacts (7). Data are essential for understanding the natural history, burdens and diversity of rare diseases, identifying gaps and the research priorities of PLWRD, and monitoring the effectiveness and safety of interventions (1). There are between 5,000 and 8,000 RDs, united in their chronicity and complexity but unique in cause, specific symptoms and natural histories (5, 8). Recent cross-national initiatives demonstrate scalable pathways: the European Health Data Space (EHDS) (9) and TEHDAS2 (10) provide federated data infrastructure enabling secure secondary use across 27 EU Member States. Barriers remain outside of Europe, related to fragmented governance, data-ownership uncertainty, and inequitable technical capacity particularly in low- and middle-income countries (LMICs). The Egyptian Digital Health 2030 initiative illustrates both opportunity and challenge (11). Despite constrained resources (12), Egypt is now building a national RD registry anchored in public-sector stewardship (13).

The Resolution urges the collection, analysis, and dissemination of data on PLWRD, disaggregated by income, sex, age, race, ethnicity, and other characteristics (3, 4). It advocates for the development and use of common definitions, standards, and indicators for rare diseases, as well as the sharing of data and best clinical practices across countries and regions.

To achieve this, we suggest the following actions:

1. Establish and support national and international registries and biobanks for rare diseases, that collect and store clinical, biological, and socio-economic data from patients and families, following local ethical and legal guidelines. In this regard, the International Rare Disease Research Consortium (IRDiRC) was established in 2011 to strengthen global collaboration in rare disease research. It unites public funders, industry partners, and umbrella patient organizations under shared goals, including the provision of diagnoses for undiagnosed individuals within one year, stimulation of the development of 1,000 new therapies, and establishing frameworks to assess their effectiveness (14).
2. Promote and facilitate data interoperability and accessibility, by using common data models, formats, and platforms, and by ensuring data protection and privacy. The implementation of rare diseases coding (i.e., Orphacodes) and ICD-11 extensions to standardize metrics in government (including health and other sectors) and research systems is critical (15).
3. Enhance data literacy and capacity among researchers, clinicians, patients, and policymakers, by providing training, tools, and guidance on data collection, analysis, and use.

## Diagnosis

A major challenge for RDs are the delays and difficulties in obtaining a correct and timely diagnosis (16). While universal genomic sequencing is feasible in high-income contexts e.g., Rady Children's Institute for Genomic Medicine achieved Medicaid coverage for rapid whole genome sequencing in 18 US states by 2025 (17). Similarly, the BeginNGS programme is piloting genome sequencing at population scale to screen newborns for over 500 rare conditions, combining genomic data with artificial intelligence (AI) to enhance diagnostic speed and precision (18). LMICs face affordability and infrastructure barriers limited bioinformatics capacity, reagent costs, and absence of trained workforce (19). Nevertheless, emerging LMIC models show promise, the iHope Programme provides free whole-genome sequencing in 21 countries, with >40% diagnostic yield and 70% of findings altering management (20). Diagnosis is crucial for accessing appropriate treatment and care, as well as for planning and coping with the condition. However, many rare diseases are complex, phenotypically heterogeneous, and poorly understood, making them hard to recognize and diagnose (16).

The Resolution calls for accelerating efforts toward the achievement of universal health coverage for all persons, including those living with a RD. It stresses the need to strengthen health systems and workforce capacity, and to ensure equitable access to quality and affordable health services and products (4).

To improve the diagnosis of rare diseases, we suggest the following actions:

1. Support the initiation and expansion of Undiagnosed Diseases Programs and their national and international networks (21).
2. Develop and implement operational definitions for undiagnosed rare diseases that can be used at different stages of the diagnostic journey to identify people who are not yet diagnosed and assess the impact of diagnosis (22).
3. Develop and implement national and international strategies and guidelines for the diagnosis of RD, that define the roles and responsibilities of different clinicians and stakeholders, and that promote the use of evidence-based and cost-effective diagnostic tools and pathways.
4. Invest and innovate in diagnostic technologies and methods, such as genomic sequencing, biomarkers, and artificial intelligence, to enhance the accuracy, speed, and affordability of diagnosis.
5. Empower and educate patients and families, by providing them with information, support, and counseling on the diagnostic process, and by involving them in decision-making and advocacy.

## Treatment

A further challenge for rare diseases is the lack of effective and accessible treatments and therapies. Equitable access to orphan drugs remains a global priority, yet drug pricing mechanisms often pit innovation against affordability (23). OECD and EU work highlights the lack of systematic monitoring of access and wide cross-country variation in availability and price-setting approaches. Furthermore 95% of rare diseases lack approved therapies and economic costs exceed USD 7 trillion annually (24, 25). This underscores the need for sustainability models linking pricing, such as managed-entry agreements (MEAs) and outcome-based reimbursement (OBR), to value including health, social and economic returns.

Sustainability models notably managed-entry agreements (MEAs) and outcome-based reimbursement (OBR) are increasingly used to share risk when evidence is immature, yet their implementation is complex, and results are mixed (26). Health Technology Assessment (HTA) bodies have adapted methods (e.g., NICE severity modifiers/HST route) (27) to recognize disease burden and uncertainty, while joint regulatory/HTA alignment such as the Access Consortium (Canada–UK–Australia–Singapore–Switzerland) aim to accelerate safe, affordable access to advanced therapies including gene and cell therapies (28).

The Resolution recognizes the right of persons living with a rare disease to enjoy the highest attainable standard of health, and to benefit from scientific progress and its applications (3, 4). It encourages the development and delivery of safe, effective, and affordable health products and services for rare diseases, and the promotion of research and innovation in this field (3).

To advance the treatment of rare diseases, we suggest the following actions:

1. Increase and diversify funding and incentives for rare disease treatment development and evaluation, by mobilizing public and private resources, and by implementing policies that reward innovation, collaboration (locally and internationally), and social impact.
2. Foster and facilitate translational and clinical research for rare diseases, by establishing and supporting research networks and infrastructures, building clinical trial readiness, and streamlining and harmonizing regulatory and ethical processes. For instance, the Prader-Willi Syndrome (FPWR) Registry enabled the first FDA approval for hyperphagia treatment by transforming patient-reported data into regulatory evidence. Participation of CDKL5 families in registries and natural-history studies facilitated subsequent clinical trials, culminating in the approval and clinical use of Ganaxolone for seizure control in CDKL5 deficiency disorder (29–31).
3. Ensure and expand access and affordability of rare disease treatments and therapies, by implementing pricing and reimbursement policies that balance innovation and sustainability, enhancing the availability and distribution of health products and services, and by understanding benefits across individual, social and economic domains.

## Inclusion

The final challenge for rare diseases is the marginalization and exclusion of PLWRD and their families from society. Participation is essential for ensuring the dignity, autonomy, and inclusion of patients and families, as well as for fostering their empowerment and resilience. However, many PLWRD face discrimination, stigma, and barriers to accessing education, employment, and social protection.

The Resolution affirms the importance of non-discrimination and social inclusion for PLWRD, and the need to respect their human rights and fundamental freedoms (3). It also urges the promotion of access to full and productive employment and decent work, along with appropriate measures for financial inclusion for PLWRD and their families (3). The Rare Care Center at Perth Children's Hospital demonstrates a rights-based care-coordination model that integrates health, education, and social services, yielding 33% reductions in bed-days and AUD $13,400 savings per child (32).

To enhance the participation of persons living with a rare disease, we suggest the following actions:

1. Raise and improve awareness and understanding of rare diseases among the general public, the media, and the policymakers, by conducting campaigns, events, and education programmes, and by disseminating accurate and reliable information and stories. Research is required to deliver innovative way of providing these interventions and assessing their impact.
2. Protect and promote the human rights and interests of persons living with a rare disease and their families, by adopting and enforcing laws and policies that prohibit discrimination and harassment, and that ensure equal opportunities and access to resources and services. Research is required to help formulate law and policy and to assess accountability and impact.
3. Improve the mental health and wellbeing of PLWRD and their families through approaches that focus on the unmet needs inherent in rarity that adds further to the burden on top of existing complexities.
4. Identify and address stigma associated with rare diseases in hospital, school, community and work settings to improve access to services and reduce inequity.
5. Develop strengths-based approaches that enable personal growth and development and recognize and build on the talents, resourceful and ingenuity of PLWRD and their families.
6. Embrace Diversity, Equity and Inclusion including to maximize participation, achieve critical mass for e.g., clinical trials and leave no one behind.
7. Focus on care coordination and integration including cross-sector (e.g., health, education, disability, social services etc.) and interagency approaches that support living the best lives possible.
8. Support and strengthen the rare disease community within civil society, by providing them with funding, capacity-building, and opportunities for representation, and by engaging them in policymaking and research.

## Conclusion

The UN Resolution on Persons Living with a Rare Disease and Their Families is an historic achievement for the global rare disease community. It provides a powerful and comprehensive framework for addressing the challenges and needs of this population, and for advancing their health and wellbeing. Research and innovation are key components in addressing the unmet needs identified in the Resolution, as they can generate new knowledge, solutions, and opportunities for PLWRD and their families. The proposed framework of Resolution-Informed Research Governance (RIRG) advances three interdependent dimensions: epistemic innovation, by re-casting the Resolution as a global research mandate that integrates human-rights obligations with scientific enterprise; operational pathways, by drawing on cross-national exemplars including the European Health Data Space, Egypt's Digital Health 2030 Strategy, and the Rare Care Center in Western Australia; and evaluative mechanisms, by delineating metrics and governance benchmarks for monitoring progress and accountability. Within this schema, the development and networking of Centers of Expertise for Rare Diseases under the auspices of the Global Network for Rare Diseases represent a pivotal implementation mechanism transforming normative commitments into measurable improvements in health outcomes, research capacity, and global equity.

However, research alone is not enough. It must be accompanied by political commitment and funding across all sectors, social mobilization, and international cooperation, to ensure that no one living with a rare disease is left behind. Accordingly, developing and globally networking Centers of Expertise for Rare Diseases under the Global Network for Rare Diseases (33) are a critical step to support health and wellbeing outcomes, including through fostering research.
